# The scope of plant direct

**DOI:** 10.1002/pld3.13

**Published:** 2017-10-17

**Authors:** Ivan Baxter

*Plant Direct* is now fully up and running and accepting manuscripts and publishing papers. We are very excited to see the great science that has been submitted. But many have asked me what our statement “We seek to be the sound science plant journal for the community of ASPB members, SEB members, and the authors who publish in our society journals” indicates about our scope—in terms of both subjects and types of papers. In this editorial, I will attempt to answer those questions in some detail.

We are hoping to fill a niche for the entire plant science community—a place for everyone to publish all of their sound science. The inaugural Editorial for *Plant Direct* provides more details about our mission as a community journal. The scope of *Plant Direct* includes all of the original research that you might submit to *Plant Physiology*,* The Plant Cell,* or *The Plant Journal*—we simply apply different review criteria and make judgments on sound science rather than impact or novelty. In addition, we are also interested in the following types of research:

*Negative results:* Do you have a research project that, while well executed, did not produce a significant result around which to frame your manuscript? For example, did you identify a set of genes—a gene family, candidates from quantitative genetics or transcript profiling experiments, and screen mutants of the genes for a given phenotype—but none were different from wild type? Those results still have value to our community, and we would be happy to publish them. You just have to convince your reviewers that you conducted the experiments rigorously and to the technical standards of the community.

*Replication Experiments:* Have you intentionally, or unintentionally, repeated an experiment that other researchers have already published? Just like with negative results, knowing that these results are or are not replicable has value to the community.

*Methods, software tools, and datasets:* These represent incredibly important components of any research project, and many times it can be useful to publish them quickly as a discreet, citable unit. *Plant Direct* is happy to provide a venue for our community to rapidly disseminate these contributions.

Functionally, the subject matter scope is defined by the members of our editorial board: An editor has to make an affirmative decision to handle a manuscript, so they need to feel that its subject matter is close enough to their expertise for them to evaluate. The effective scope will expand as our board grows. To give you a feel for the current board, our editors selected keywords representing their expertise. You can find a full listing of the keywords and associated editors here, and the figures represent the breadth of the terms they used. However, authors should not consider these lists to strictly define our scope; we are happy to consider manuscripts on subjects related to our expertise—for example, work on plant species that are not listed.

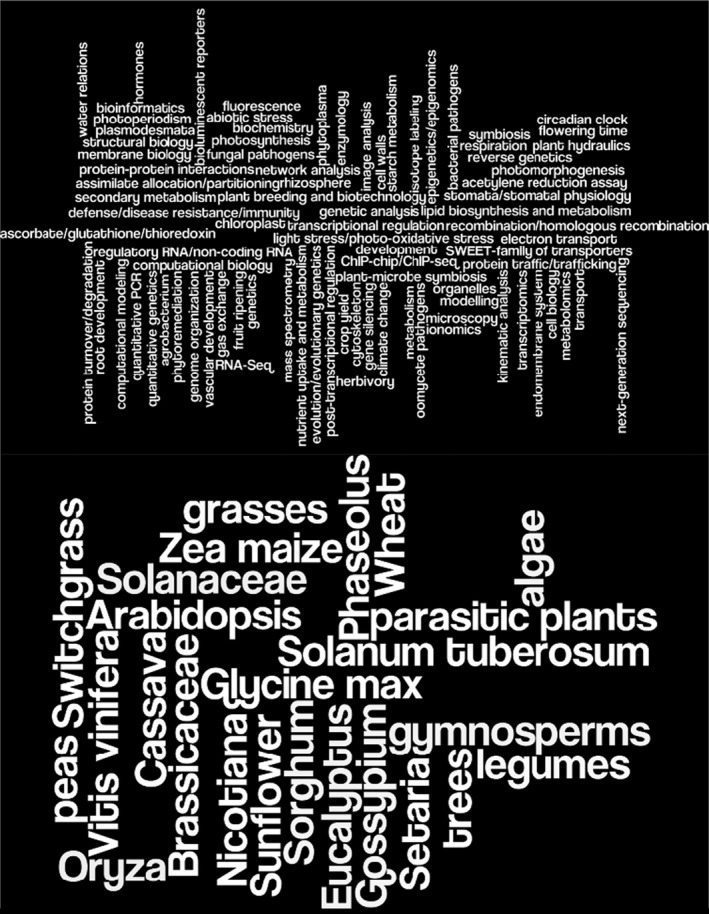

I hope that this editorial has answered your questions about whether *Plant Direct* is the right place for your next manuscript. But if you still have questions or concerns, please do not hesitate to contact us at PlantDirect@wiley.com.

